# OAB score: A clinical model that predicts the probability of presenting overactive detrusor in the urodynamic study

**DOI:** 10.1590/S1677-5538.IBJU.2017.0213

**Published:** 2018

**Authors:** Leandro Cristian Arribillaga, Marta Ledesma, Ariel Montedoro, Florencia Pisano, Rubén Guillermo Bengió

**Affiliations:** 1 Centro Urológico Profesor Bengió, Córdoba, Argentina

**Keywords:** Urinary Bladder, Overactive, Urodynamics

## Abstract

**Purpose:**

To create a predictive model of involuntary detrusor contraction (IDC) to improve the diagnostic accuracy of overactive detrusor (OAD), associating overactive bladder (OAB) symptoms with other clinical parameters in the female population.

**Materials and Methods:**

A total of 727 women were studied retrospectively. In all of them, urodynamic study was conducted for urogynecological causes. Demographics information, personal history, symptoms, physical exam, a 3-day frequency/volume chart and urinary culture, were collected in all patients and they subsequently underwent uroflowmetry and urodynamic studies. A logistic regression model was performed in order to determine independent predictors of presence of IDC. Odd ratio (OR) estimation was used to assign a score to each one of the significant variables (p≤0.05) in the logistic regression model. We performed a ROC curve in order to determine the predictive ability of the score in relation to the presence of OAD.

**Results:**

presence of OAD was evident in 210 women (29%). In the logistic regression analysis, independent predictors of OAD were urgency, urgency incontinence, nocturia, absence of SUI symptoms, diabetes mellitus, reduction of vaginal trophism and bladder capacity below 150 mL. The probability of IDC diagnosis increases as the score raises (Score 0: 4% until Score ≥10: 88%). Sensitivity was 71% and specificity 72%. The area under the curve of OAB score was 0.784 (p>0.001).

**Conclusions:**

OAB score is a clinical tool that shows higher diagnostic accuracy than OAB symptoms alone to predict overactive detrusor.

## INTRODUCTION

Overactive Bladder (OAB) is a complex disorder that affects an important group of people, altering their quality of life (1); even to a greater extent than other chronic diseases such as hypertension and diabetes (2). It is a medical entity widely predominant, affecting about 10.7% of the population worldwide (3).

The consensus of the International Urogynecological Association (IUGA) and the International Continence Society (ICS) defines this syndrome as the presence of urgency with or not urge urinary incontinence (UUI), usually associated with an increase of frequency and nocturia in the absence of urinary infection or other underlying pathology (4). This concept implies that the term OAB does not define clearly the etiology. Traditionally, this syndrome has been associated with the presence of involuntary detrusor contractions (IDC) in the filling phase of the cystomanometry; this urodynamic observation is called overactive detrusor (OAD). IDC identification in the urodynamic study in patients with OAB occurs in around 50% of patients (5), creating controversies among several authors of the initial diagnostic algorithm (6, 7). Since the definition of OAB in 2002, there is little evidence that symptoms that belong to this syndrome could diagnose with accuracy the presence of OAD (8). Therefore, the association between OAB and OAD is not yet clear. Taking into account the above-mentioned information, the creation of a model that improves the predictive ability of OAB symptoms by itself is essential; this system should complement them with other clinical variables with the aim of improving the identification of IDC and in this way, reduce the need of unnecessary studies.

The purpose of this study is to create a predictive model of IDC (OAB score) in order to improve the diagnostic accuracy of OAD associating OAB symptoms with other clinical parameters in the female population.

## MATERIALS AND METHODS

A total of 770 women were studied retrospectively in whom urodynamic study was conducted for urogynecological causes at Centro Urológico Profesor Bengió between January of 2010 and January 2013. Patients with history of urethral stricture, neurological, bladder or colonic disease, pelvic radiotherapy, anticholinergic use and medication that could alter bladder function were excluded from the study.

The study population included 727 female patients. Demographic data was collected from all patients, such as previous surgical procedures and diseases and lower urinary tract symptoms. Subsequently, a complete physical exam was carried out evaluating vaginal trophism, urethral mobility (Q tip), cough stress test and Valsalva maneuver with full and empty bladder to determine the presence or not of stress urinary incontinence (SUI), and evaluation of the presence of pelvic organ prolapse with empty bladder according to Pelvic Organ Prolapse Quantification system (POP-Q).

In all patients, a 3-day frequency/volume chart was requested and urinary culture should be negative. All urodynamics (UD) were carried out with Ecud Compact^®^ equipment according to ICS protocol of good urodynamic practice (9). Initially, an uroflowmetry was performed with ACE software in a private environment and micturition volume, maximum flow rate (Q_max_) and post void residual urine volume (PVR) measured by catheterization (considered abnormal >20% of micturition volume) were recorded. The Multichannel urodynamic study was carried out with the patient in both positions standing up and seated, at an irrigation speed of 30 mL/min, with water at ambient temperature (25°C) and filling towards voiding intense desire or 500 mL irrigation. During the filling cystometry, sensitivity, compliance, bladder capacity and presence of IDC spontaneous or provoked were observed.

Urine incontinence by cough stress tests with Valsalva maneuver every 100 mL was studied. Lastly, a voiding cystometry (pressure/flow curve) was carried out.

Patients were divided into two groups according to the presence or not of overactive detrusor. The variables evaluated were age, parity, symptoms such as urgency, UUI, nocturia (> one night micturition), frequency (>7 daytime micturition), SUI according to IUGA/ICS definitions, voiding alterations: stream, hesitancy and incomplete voiding sensation; prolapse presence (bulk or vaginal weight) and recurrent UTI (+ 2 annual infections), hysterectomy and other pelvic surgeries, menopause. During the physical exam, vaginal trophism, anterior, apical and posterior prolapse were evaluated. The variables studied in uroflowmetry were QMax and PVR. To conclude, bladder capacity was determined according to the frequency/volume chart.

In all patients, using the chi-square statistical method, significant variables (p≤0.05) for OAD were entered in a logistics regression model in order to determine independent predictors for the presence of involuntary detrusor contractions. OR unity value was used to assign a score to each one of the significant variables (p≤0.05) in the regression logistics model. The sum of these in each patient determines a score (varying from 0 to 10). In the end, we performed a ROC curve in order to evaluate the ideal score cut-off point, determining its sensitivity and specificity; the predictive capacity of the score in relation to the OAD presence was determined by the area below the curve.

## RESULTS

The mean age of the studied population was 60.5 years old (23-89). The presence of OAD was demonstrated in 210 women (29%). Other diagnostic findings were urodynamic SUI in 74% of the cases, prolapse 35%, voiding alterations in 25% and 10% of UTI recurrence. In Table-1 we demonstrate the clinical and urodynamic comparison between patients with and without OAD.

**Table 1:** Comparison of clinical and urodynamic profile of patients with and without OAD.

Variable		No OAD (n:517)	OAD (n:210)	OR	P
Age > 60 year old		270 (52.2%)	129 (61.4%)	1.46	0.02
Parity ≤ 1		114 (22.1%)	52 (24.8%)	1.16	0.42
Parity ≥ 2		403 (77.9%)	158 (75.2%)	1.21	0.43
**Symptoms**					
	Urgency	321 (62.1%)	185 (88.1%)	4.5	<0.001
	UUI	249 (48.1%)	167 (79.5%)	4.2	<0.001
	Nocturia	126 (24.4%)	141 (67.2%)	6.3	<0.001
	Frequency	80 (15.5%)	93 (44.3%)	4.3	<0.001
	Voiding alterations	121 (23.4%)	58 (27.6%)	1.25	0.23
	POP	189 (36.5%)	69 (32.9%)	0.85	0.34
	UTI recurrence	46 (8.9%)	29 (13.8%)	1.64	0.04
	Absence of SUI	107 (20.7%)	85 (40.5%)	2.6	<0.001
**Background**					
	Hysterectomy	129 (24.9%)	58 (27.6%)	1.15	0.45
	Pelvic Surgery	68 (13.1%)	30 (14.3%)	1.10	0.68
	Menopause	396 (76.6%)	171 (81.4%)	1.34	0.15
	Diabetes	21 (4%)	19 (9.1%)	2.35	0.007
**Physical exam**					
	Vaginal Trophism Reduction	215 (41.6%)	120 (57.1%)	1.87	0.001
	Presence of SUI	205 (39.6%)	66 (31.4%)	1.43	0.05
	Anterior POP	169 (39.7%)	59 (28.1%)	0.80	0.22
	Middle POP	76 (14.7%)	40 (19.1%)	1.37	0.14
	Posterior POP	85 (16.4%)	49 (23.3%)	1.55	0.02
Bladder capacity <150 mL in frequency/volume chart		60 (11.6%)	57 (27.1%)	2.84	<0.001
**Uroflowmetry**					
	Q_max_ <20 mL/sec	67 (12.9%)	30 (14.3%)	1.12	0.63
	High PVR	45 (8.7%)	24 (11.4%)	1.35	0.25

In the univariate analysis, we could demonstrate that factors that predispose the presence of OAD were: age >60 years old, urgency, UUI, nocturia, frequency, UTI recurrence, absence of SUI symptoms, diabetes, reduction of vaginal trophism, POP posterior and bladder capacity <150 mL in the frequency/volume chart. These variables were included in the multivariate model observed in Table-2.

**Table 2:** Multivariate analysis of OAD clinical predictors.

Variable	OR	CI 95%	P
Age > 60 years old	0.73	0.46-1.17	0.19
Urgency	1.56	1.04-2.66	**0.05**
UUI	1.97	1.08-3.60	**0.02**
Nocturia	3.46	2.21-5.42	**<0.001**
Frequency	1.33	0.83-2.13	0.24
UTI recurrence	1.09	0.61-1.93	0.77
Absence of SUI symptoms	2.33	1.50-3.64	<**0.001**
Diabetes	2.53	1.20-5.33	**0.01**
Vaginal Trophism reduction	1.59	1.01-2.38	**0.05**
POP posterior	1.45	0.91.2.29	0.11
bladder capacity <150 mL	2.12	1.20-3.08	**0.006**

In the logistics regression analysis, we observed that independent predictors for OAD are urgency, UUI, nocturia, absence of SUI symptoms, presence of diabetes, reduction of vaginal trophism and bladder capacity below 150 mL. In Table-3 we assigned the result of the score in relation to OR.

**Table 3:** Variable score according to OR logistics regression analysis.

Variable	Point Score
Nocturia	**3**
Vesical capacity <150 mL	**2**
Absence of SUI symptoms	**2**
Diabetes	**2**
Urgency	**1**
UUI	**1**
Vaginal Trophism reduction	**1**

Table-4 presents the OAB score, where for each result obtained by the sum of parameters in each patient, it is stablished the possibility of presenting OAD.

**Table 4:** OAB Score and IDC probability in the urodynamic study.

Score	IDC Probability
0	4%
1	8%
2	20%
3	16%
4	24%
5	29%
6	45%
7	61%
8	65%
9	86%
≥10	88%

**Figure 1:**
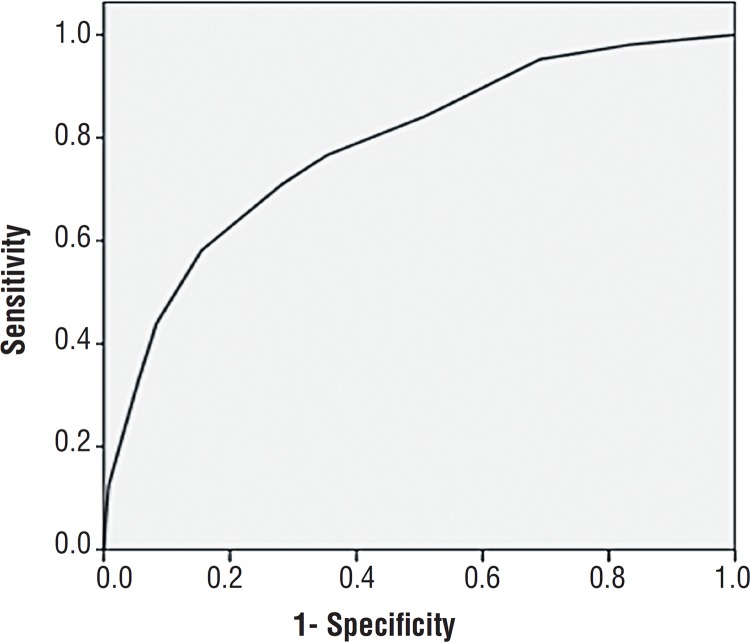
OAD and OAB Score ROC curve.

As we have demonstrated, as the score increases, the probability for presenting IDC in the urodynamic study increases as well starting at 4% with Score 0, reaching 88% with Score ≥10. If we only include the presence of symptoms of OAB (urgency, UUI, nocturia and frequency). the sensitivity is 57.4%, the specificity 83.7% and the area under the curve 0.70. After the ROC curve was drawn we determined that the ideal cut-off point of the Score is 5, with a sensitivity of 71% and specificity of 72%. In Figure-1 we point out that the area under the curve in order to determine the predictive capacity of the OAB score is 0.784 (p<0.001).

## DISCUSSION

Overactive bladder syndrome can affect significantly quality of life. Therefore, its etiology and an accurate clinical diagnosis have been important goals over the time. The exact definition for ICS refers to a syndrome where the principal symptom is urgency, with or without UUI, usually associated to frequency and nocturia. Although according to the recommendation of the International societies (ICS/IUGA), the urodynamic study is not routinely necessary in patients with OAB, the cystometric evaluation, as part of the clinical and diagnostic evaluation, is widely accepted as the gold standard in the evaluation of low urinary tract dysfunctions, including OAD (10); the diagnosis of OAD is based on the presence of involuntary detrusor contractions in the filling and storage phases in women with OAB symptoms (4, 8). Thus, many professionals believe that a correct diagnosis cannot be determined in some patients if urodynamic is not carried out and therefore will not receive a proper treatment, considering that the bladder has been described as an unreliable witness (11). Nevertheless, the reproduction of OAB symptoms with IDC presence in the urodynamic study is not yet clear. In our population with urgency, the presence of OAD is 35% and it can rise to 57% if we add other OAB symptoms (UUI, nocturia and frequency). Several previous series have documented an incidence of OAD that ranges between 32-54% in women with OAB (5, 7, 12, 13). At the same time, the routine use of ambulatory urodynamic study raises the diagnosis to 70% (14); although it is also important to point out the presence of OAD in 68% of voluntary asymptomatic women, limiting its use as a first line practice (15). However, these variations can be subject to different factors that may affect the detection of OAD, including speed in filling, position of the patient and provocation maneuvers (16). This shows the need of a more accurate clinical model to reduce unnecessary urodynamic investigations from the diagnostic point of view with higher morbidity (invasiveness).

Colli et al. carried out a revision of 20 studies (1980-2000), evaluating sensitivity and specificity of OAD in patients with OAB symptoms, finding a sensitivity of 0.69 (0.36-0.96) and a specificity of 0.60 (0.21-0.97) to predict IDC presence based on symptoms (17). This data is in accordance to our series.

In order to improve the ability to predict DHA along with symptoms of OAB (except urinary frequency), other clinical variables were analyzed by a multivariate model of logistics regression (bladder function capacity <150 mL in 3 day frequency/volume chart, absence of SUI symptoms, diabetes and vaginal trophism reduction). Urinary frequency was excluded since it was not an independent predictor of OAD, similarly to previous studies, without statistical signifance (7, 18, 19).

OAB Score was proposed to improve the diagnostic capacity in patients with only OAB symptoms (sensitivity 71%, specificity 72% and ROC curve 0.78), perhaps creating a clinical tool that predicts more accurately the presence of IDC.

Some previous observations have included other signs and symptom to improve predictability of OAD (5, 15). In our studied population, we found light differences in the age of patient with or without OAD, although it was not expressed as an independent predictor as in previous reports, where it was observed 3-5 years of differences in patients with presence of IDC (13, 20). Harris and Haylen demonstrated that nulliparity increased the risk of IDC, which was not concurrent to our findings (18, 21). In the same manner that our model series, it was previously demonstrated that the absence of SUI has a high possibility of OAD (18, 22). Currently, there are controversies on the risk of OAD in relation to POP presence, hysterectomy and previous pelvic surgery, although in our series this was not demonstrated as a variable to be considered statistically significant (12, 23).

Previous studies did not demonstrate variable differences in daytime or nocturnal frequency in the frequency/volume chart; however, women with IDC presence have a lower functional capacity in some preceding series (12, 13).

Diabetes mellitus (DM) is another independent risk factor in our series. Epidemiological study has shown that DM is an independent factor for OAB (24).

Just as in our study, several authors have shown that vaginal atrophy due to estrogen insufficiency is a recognized cause or contributory factor in overactive bladder (25, 26).

Two previous publications have tried to improve clinical diagnosis of OAD by predicting models. Vella et al. described a Score after a clinical survey of 171 women that included 9 symptoms, improving the sensitivity and specificity; yet it is not clear whether their selected study model was univariate or multivariate (27). Haylen et al. improves the prediction of OAD adding to the classical OAB symptoms (except urinary frequency), the lower presence of parity (0-1), absence of SUI and absence of POP signs, though such improvement was minimum and would not be of great clinical utility (ROC curve 0.70 vs. 0.74) (18).

In our study, the proposal of an OAB Score was based in the construction of a clinical model that generated more accuracy in OAD diagnosis, given that if this diagnosis is inappropriate, it can lead to bad medical treatments, while the possibility of relieving the patient from its symptoms is less probable. Also, it could reduce the need of urodynamic studies and introduce rapidly first line therapies in OAB, particularly conservative behavioral measures and medications. Therefore, our score could be very useful in primary practice when managing patients with OAB, since it can be reproduced easily, improve cost-effectiveness and reduce the use of unnecessary treatment that generate disappointment to the patient, with higher morbidity and adverse events that worsen even more quality of life.

## CONCLUSIONS

OAB score is a clinical tool with higher diagnostic precision than OAB symptoms alone to predict OAD. It is easily reproducible as is based on clinical parameters commonly used in the daily urogynecological practice.
